# Hepatic and renal histopathological changes in lambs fed sisal mucilage silage with or without moisture-absorbing additives

**DOI:** 10.1007/s11250-026-05344-8

**Published:** 2026-09-21

**Authors:** Vitor Marques de França, Manoel Francisco de Sousa, Tomás Guilherme Pereira da Silva, João Vitor Fernandes Clemente, Emanuel Felipe de Oliveira Filho, Valdemiro Amaro da Silva Júnior, Paulo Sérgio de Azevedo, Edson Mauro Santos, Francisco Fernando Ramos de Carvalho, Adriana Guim, Pierre Castro Soares

**Affiliations:** 1 Department of Veterinary Medicine, Federal Rural University of Pernambuco, Dom Manuel de Medeiros Street, s/n, Dois Irmãos, Recife, PE 52171-900 Brazil; 2 Brazilian Agricultural Research Corporation (EMBRAPA) - Cotton, Oswaldo Cruz Street, 1143, Centenário, Campina Grande, PB 58428-095 Brazil; 3 Federal Institute of Education, Science and Technology of Sergipe (IFS), Propriá Campus, Rodovia BR 101, Km 5, s/n, Centro, Propriá, SE 64750-000 Brazil; 4 Department of Animal Science, Federal Rural University of Pernambuco, Dom Manuel de Medeiros Street, s/n, Dois Irmãos, Recife, PE 52171-900 Brazil; 5 Barra Multidisciplinary Center, Federal University of Western Bahia, Av. 23 de Agosto, s/n, Assunção, Barra, BA 47100-000 Brazil; 6 Department of Animal Science, Federal University of Paraíba, Rodovia PB 79, Km 12, s/n, Bairro Universitário, Areia, PB 58397-000 Brazil

**Keywords:** *Agave sisalana*, Ensilage, Oxalates, Saponins, Small ruminants, Tissue lesions

## Abstract

This study evaluated the effects of diets containing sisal mucilage silage, with or without moisture-absorbing additives, on the hepatic and renal histopathological profiles of lambs. Eighteen Soinga lambs (average initial body weight of 19 ± 1.38 kg) were allocated in a completely randomized design among three diets: sisal mucilage silage without additive (SMS); sisal mucilage silage ensiled with wheat bran (SMS-WB); and sisal mucilage silage ensiled with ground corn (SMS-GC). The experimental period lasted 60 days, after 14 days of adaptation to the diets. SMS without additives induced severe hepatic coagulative necrosis and microvesicular steatosis, alongside moderate-to-severe renal proliferative glomerulonephritis and glomerular atrophy. SMS-WB acted as a potent hepatoprotector, completely preventing macrovesicular steatosis and pericentral necrosis while down-grading microvesicular steatosis to a mild degree. In the kidneys, SMS-GC provided the most effective glomerular protection, mitigating both proliferative glomerulonephritis and glomerular atrophy to mild intensities. On the other hand, SMS-WB reduced only glomerular atrophy to a mild level, failing to contain proliferative glomerulonephritis. Renal medullary dystrophic calcification remained unaltered across all treatments (66.6%), indicating an intrinsic lesion profile of sisal mucilage ingestion. In conclusion, while sisal mucilage silage exhibits intrinsic hepatorenal toxicity in lambs, the use of moisture-absorbing additives is a viable mitigation strategy. Wheat bran serves as an effective hepatoprotector, whereas ground corn provides superior glomerular preservation. Therefore, the strategic inclusion of these additives is crucial to enable the safe use of sisal by-products in ruminant nutrition without compromising animal health.

## Introduction

In semiarid regions worldwide, small ruminant production is constrained by seasonal forage availability, as irregular rainfall and prolonged droughts reduce both the availability and nutritional quality of native pastures. To mitigate this problem, the use of locally available agro-industrial by-products has emerged as a sustainable and economically viable alternative for livestock feeding (Zamudio et al. 2009; Nudda et al. 2025; Marcos et al. 2026).

In this context, sisal mucilage (*Agave sisalana* Perrine), a by-product generated in large quantities during fiber decortication, represents a promising feed resource. When preserved as silage, it may efficiently supply both water and energy requirements of finishing lambs (Zamudio et al. 2009; Santos et al. 2011; Souza et al. 2018). Sisal fiber decortication generates substantial amounts of waste, with commercial fibers accounting for only 3% to 5% of the total leaf weight, while the remaining 95% constitutes the mucilage. Furthermore, due to its massive production volume, mucilage frequently accumulates at processing sites, where it is either discarded into the environment or fed directly to livestock (Silva and Beltrão 1999). This by-product consists primarily of pulp characterized by a high moisture content and a significant fraction of non-fibrous carbohydrates (NFC) (Brandão et al. 2011; Souza et al. 2018; Silva 2020; Ferraz-Almeida et al. 2025).

The use of agro-industrial by-products as moisture-absorbing additives aims to increase dry matter (DM) content, thereby improving the fermentation profile and nutritional value of silages (Bonfá et al. 2020; Guimarães et al. 2023). When evaluating the effects of different additives on silage produced from the wet residue of sisal decortication, Brandão et al. (2011) observed that most inclusion materials successfully increased both the DM and crude protein contents of the resulting silages.

Some studies have shown the presence of secondary metabolites in *Agave* plants, including oxalates, flavonoids, and saponins (Salinas et al. 2001; Chen et al. 2009; Pérez et al. 2013; Sidana et al. 2016). Although these bioactive compounds may exhibit beneficial properties at low doses, high dietary inclusion levels can induce toxicity, mucosal irritation, or systemic overload, particularly affecting metabolic and excretory organs. Mendes et al. (2016) evaluated the toxicity of sisal waste extracts on the reproductive organ weights and testicular tissue of adult rats, reporting that these extracts altered organ mass and induced gonadotoxic effects.

The liver serves as the primary organ for the metabolism and detoxification of xenobiotics as well as endogenous and exogenous toxins, while the kidneys constitute the main route for metabolite excretion (Walling et al., 2009). Thus, histopathological lesions in these organs stand as reliable bioindicators of dietary toxicity. Although most studies on sisal mucilage have focused strictly on evaluating the productive responses of ruminants, information regarding the tissue-level impact of diets containing this strategic feed resource remains scarce.

Under the hypothesis that the use of silage additives, by diluting the inclusion rate of sisal mucilage, could mitigate potential tissue toxicity, the objective of this study was to evaluate the hepatic and renal histopathological profiles of lambs fed diets containing sisal mucilage silage, with or without moisture-absorbing additives.

## Materials and methods

### Ethical principles

This study was approved by the Committee of Ethics in the Use of Animals of the Federal Rural University of Pernambuco (UFRPE), Brazil, under protocol number 103/2017.

### Experimental site, animals, experimental design, and dietary treatments

The experiment was conducted at the Sheep Sector of the Department of Animal Science at UFRPE (Recife, Pernambuco, Brazil) at coordinates 8º04’03’’S and 34º55’00’’W. Eighteen Soinga lambs (5 months old; average initial body weight of 19 ± 1.38 kg) were allocated into a completely randomized design with three treatments and six replications per treatment. The animals were housed in individual stalls with dimensions of 1.0 × 1.2 m, provided with drinkers and feeders, and arranged in a covered sheepfold, with free access to water. The experimental period lasted a total of 74 days, comprising a 14-day adaptation period to the facilities, diets, and management, followed by 60 days dedicated to data collection and sample evaluations.

Prior to the experimental period, the lambs were identified, weighed, vaccinated against clostridial diseases (Biovet^®^ Resguard Multi, São Paulo, Brazil), dewormed (Ripercol Oral^®^, Zoetis, São Paulo, Brazil), and treated with an organic modifier (Bravet^®^, Bravet Saúde Animal, São Paulo, Brazil).

The dietary ingredients included sisal (*Agave sisalana* Perrine) mucilage silage without additive SMS (SMS; with or without additives), Tifton-85 hay (*Cynodon* spp.), soybean meal, wheat bran, ground corn, and mineral mix (Table 1).

**Table 1:** Chemical composition of ingredients of experimental diets (g/kg dry matter)

Ingredients	DM^a^	OM^b^	Ash	CP^c^	EE^d^	NDFap^e^	NFC^f^
Sisal mucilage silage without additives	216.0	854.6	145.4	80.1	47.6	222.0	470.0
Sisal mucilage silage with wheat bran	382.0	900.9	99.1	118.0	45.8	277.6	421.2
Sisal mucilage silage with ground corn	386.8	928.7	71.3	86.5	44.8	190.5	578.8
Tifton hay	864.7	926.9	73.1	52.2	21.2	679.1	130.6
Soybean meal	890.9	927.5	72.5	474.0	22.4	99.4	306.6
Wheat bran	883.9	956.2	43.8	150.0	44.9	348.0	366.5
Ground corn	892.3	983.7	16.3	88.7	44.2	108.0	719.4
Mineral mix^*^	1000.0	-	-	-	-	-	-

^a^g/kg as fed; ^b^organic matter; ^c^crude protein; ^d^ether extract; ^e^neutral detergent fiber corrected for ash and protein; ^f^non-fiber carbohydrates. ^*^1000 g contains: Ca, 173 g/kg; P, 30 g/kg; Na, 148 g/kg; Mg, 70 g/kg; Fe, 2200 mg/kg; Co, 140 mg/kg; Mn, 3690 mg/kg; Zn, 4700 mg/kg; I, 61 mg/kg; Se, 45 mg/kg; S, 12 g/kg; F, 700 mg/kg and vehicle q.s. 1000 g

The experimental diets were formulated to meet the nutritional requirements for a weight gain of 200 g/day according to NRC (2007) recommendations, in a 50:50 roughage: concentrate ratio: (1) sisal mucilage silage without additive (SMS); (2) sisal mucilage silage ensiled with wheat bran (SMS-WB); and (3) sisal mucilage silage ensiled with ground corn (SMS-GC) (Table 2).

**Table 2:** Ingredients proportion and chemical composition of the experimental diets

Ingredients (g/kg dry matter)	Treatments		
	SMS^a^	SMS-WB^b^	SMS-GC^c^
Sisal mucilage silage without additives	450	0	0
Sisal mucilage silage with wheat bran	0	450	0
Sisal mucilage silage with ground corn	0	0	450
Tifton hay	150	150	150
Soybean meal	130	80	120
Wheat bran	95	85	85
Ground corn	160	220	180
Mineral mix^*^	15	15	15
Chemical Composition (g/kg dry matter)			
Dry matter (g/kg as fed)	369.7	555.8	560.4
Organic matter	893.0	916.5	926.7
Ash	107.0	84.0	73.3
Crude protein	133.9	131.1	132.4
Ether extract	39.0	39.0	37.8
NDFap^d^	265.0	288.0	248.5
Non-fiber carbohydrates	421.0	423.0	477.5

^a^sisal mucilage silage without additives; ^b^sisal mucilage silage with wheat bran; ^c^sisal mucilage silage with ground corn; ^d^neutral detergent fiber corrected for ash and protein. ^*^1000 g contains: Ca, 173 g/kg; P, 30 g/kg; Na, 148 g/kg; Mg, 70 g/kg; Fe, 2200 mg/kg; Co, 140 mg/kg; Mn, 3690 mg/kg; Zn, 4700 mg/kg; I, 61 mg/kg; Se, 45 mg/kg; S, 12 g/kg; F, 700 mg/kg and vehicle q.s. 1000 g

Fresh sisal mucilage was obtained during the industrial processing of *Agave sisalana* leaves in Barra de Santa Rosa, Paraíba, Brazil. After collection, the mucilage was passed through a manual rotary sieve (Embrapa^®^, Rotary Sieve for Sisal Mucilage Utilization, Brazil) to remove residual fibers remaining after leaf defibration. For additive-treated silages, wheat bran or ground corn was mixed with the mucilage at a 75:25 ratio (fresh matter basis) to increase dry matter content and improve fermentation. The mixtures were ensiled in 200-kg plastic drums, manually compacted to minimize residual oxygen, hermetically sealed, and stored for 35 days before being opened and fed to the lambs.

The diets were offered twice daily at 08:00 and 15:00 h as a total mixed ration (TMR). Prior to the morning feeding, orts were collected and weighed to determine voluntary intake. The amount of feed provided was adjusted daily according to each lamb’s voluntary intake, ensuring a target refusal rate of approximately 15% on a DM basis.

### Chemical composition of feeds

The contents of DM (method 967.03), ash (method 942.05), crude protein (CP; method 981.10), and ether extract (EE; 920.29) were determined, according to the AOAC (2012). For the determination of neutral detergent fiber (NDF) the methodologies described by Van Soest et al. (1991) were used. Corrections of NDF for ash and protein (NDFap) were performed according to methodology described by Mertens (2002) and Licitra et al. (1996), respectively. Non-fiber carbohydrates (NFC) were estimated according to Hall (2000).

### Collection of hepatic and renal samples

At the end of the experimental period (day 60), All lambs were weighed to determine their final body weight and subsequently subjected to a 16-hour feed fast. The animals were then humanely slaughtered following standard animal welfare regulations and humanitarian slaughter guidelines (Brasil 2000). For the histopathological analysis, liver and kidney fragments immediately collected, as described by Silva et al. (2021) and Usman et al. (2022), respectively.

### Histopathological processing and examination

The samples were processed and analyzed at the Animal Pathology Laboratory of the Department of Veterinary Medicine at UFRPE, according to the methods described by Behmer et al. (2003). Organ fragments were fixed in 10% phosphate-buffered formalin, dehydrated in a graded series of alcohol, and cleared in xylene prior to paraffin embedding. Paraffin blocks were sectioned at a thickness of 5 μm using a manual microtome (Leica RM2125RT^®^, Germany) and stained with hematoxylin and eosin (H&E). Histopathological evaluation was performed using a light microscope (Olympus^®^ BX41, Japan), with histological images captured using digital image software (Leica ICC50^®^).

### Data evaluation

Histopathological lesions were described and presented as frequency distributions per treatment group and organ. Histopathological alterations were evaluated qualitatively by treatment group, and the severity of the primary lesions was scored as mild (+), moderate (++), or severe (+++).

## Results

**Fig. 1:**
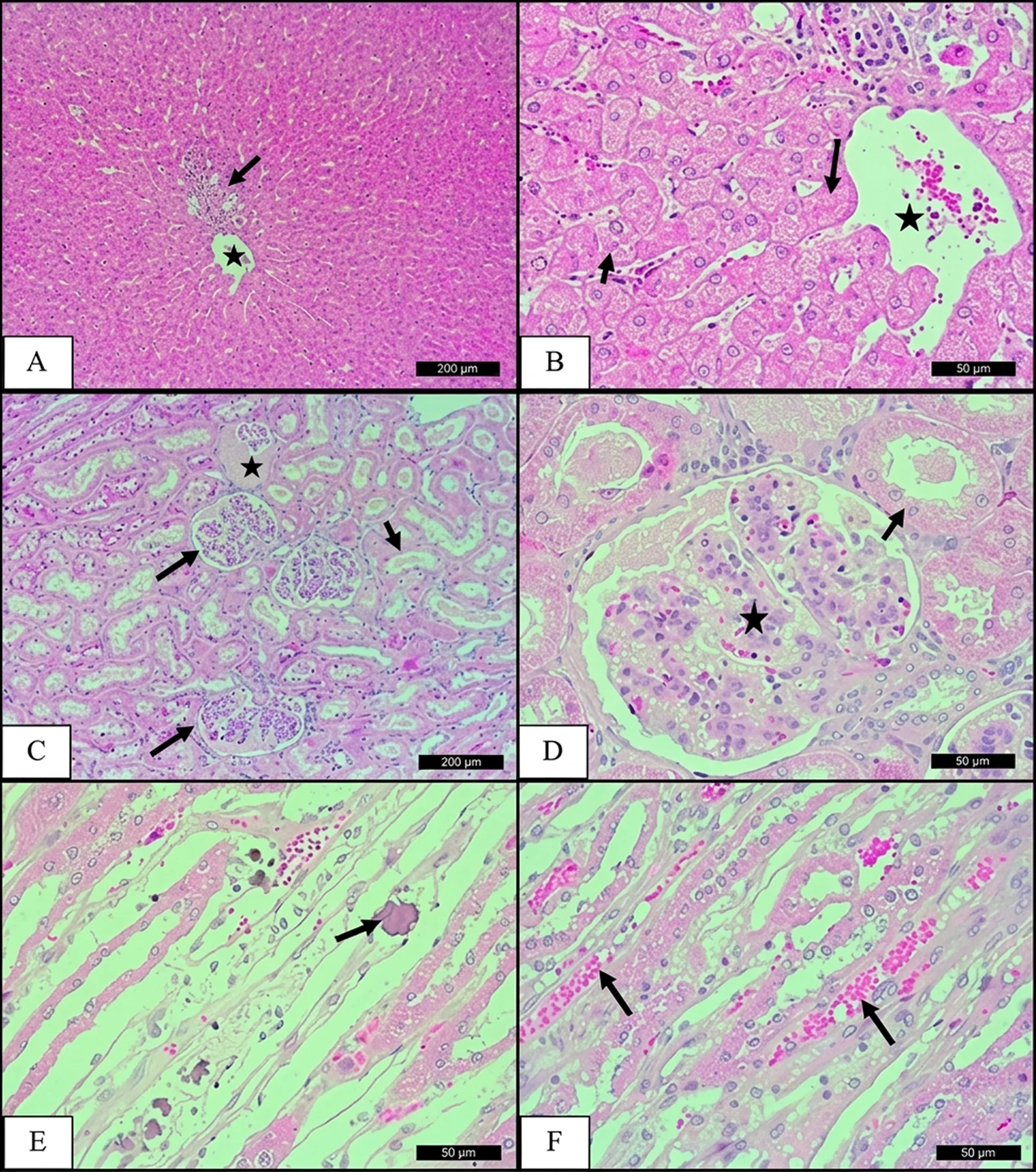
Photomicrographs of liver and kidneys parenchyma of Soinga lambs fed sisal mucilage silage without moisture-absorbing additives. (**A**) Liver: Centrilobular region showing cords of hepatocytes with varying degrees of vacuolation and cytoplasmic eosinophilia surrounding the central vein (star). Note a mild mononuclear inflammatory infiltrate, predominantly plasmacytic, adjacent to the central vein (arrow) [H&E]. (**B**) Liver: High-magnification view showing necrotic hepatocytes (large arrow) near the central vein (star). Ballooning degeneration of hepatocytes is evident, characterized by peripherally displaced nuclei and intra-cytoplasmic accumulation of eosinophilic material suggestive of Mallory-Denk bodies (small arrow) [H&E]. (**C**) Kidney: Atrophic and degenerating glomeruli (star) containing eosinophilic cellular debris within Bowman’s space (large arrows). Dilated renal tubules with intraluminal debris are also observed (small arrow) [H&E]. (**D**) Kidney: High-magnification view of membranoproliferative glomerulonephritis with early atrophy and deposition of eosinophilic material in Bowman’s space (star). Diffuse hydropic degeneration and dilation of renal tubules with intraluminal proteinaceous material are present [H&E]. (**E**) Kidney: Detailed view of dystrophic calcification (mineralization) within the medullary region [H&E]. (**F**) Kidney: Severe congestion within the medullary region [H&E]

**Fig. 2:**
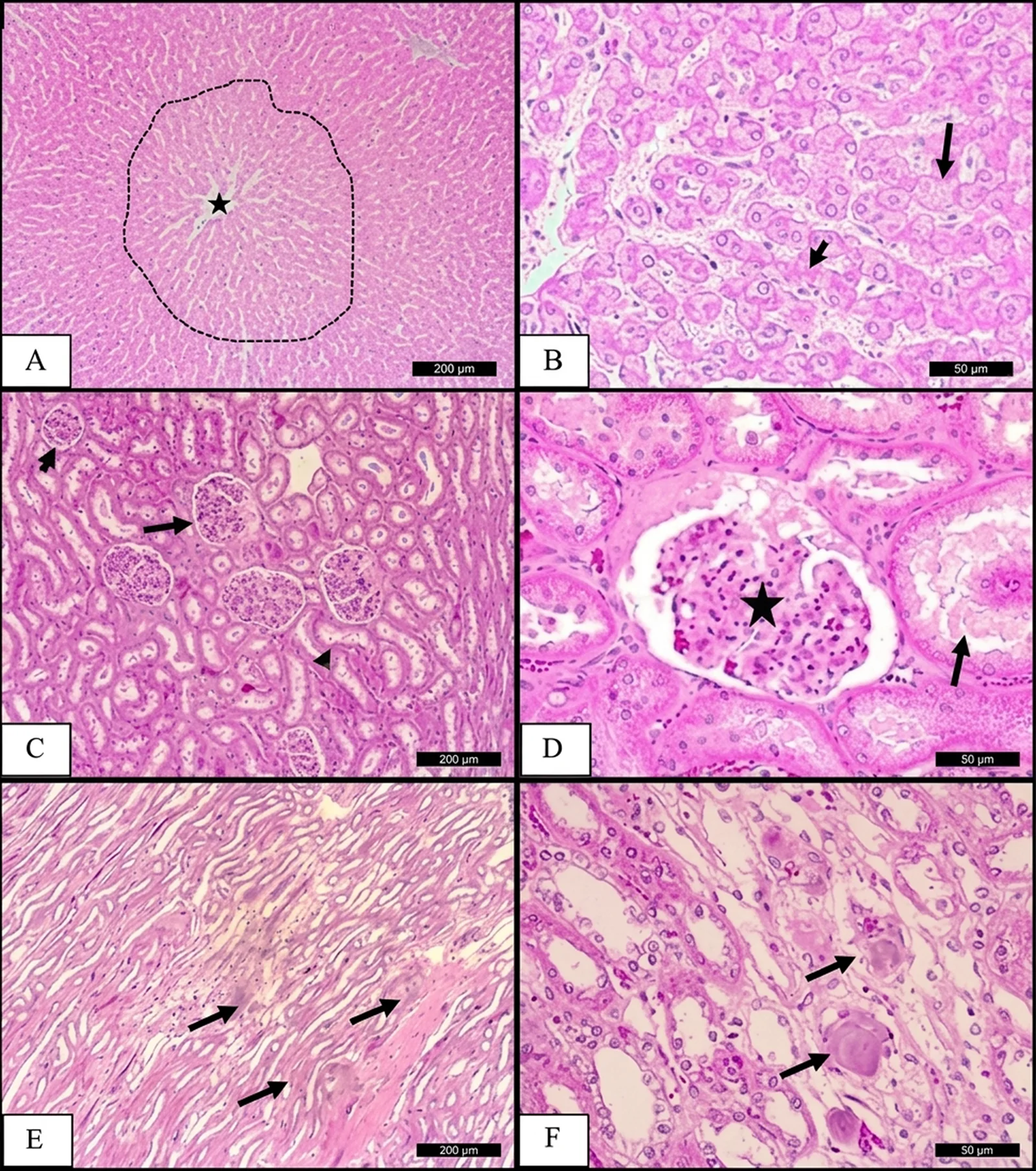
Photomicrographs of liver and kidneys parenchyma of Soinga lambs fed sisal mucilage silage with wheat bran additive. (**A**) Liver: Centrilobular region showing cords of hepatocytes with varying patterns of cytoplasmic eosinophilia surrounding the central vein (star). Note a demarcated area containing paler hepatocytes [H&E]. (**B**) Liver: High-magnification view showing necrotic hepatocytes with peripherally displaced nuclei (large arrow). Mallory-Denk bodies (hyaline degeneration) are evident [H&E]. (**C**) Kidney: Atrophic glomeruli (small arrow) alongside proliferative glomerulonephritis and accumulation of eosinophilic material within Bowman’s space (large arrow). Dilated renal tubules containing intraluminal cellular debris are also observed (arrowhead) [H&E]. (**D**) Kidney: High-magnification view of an atrophic glomerulus with eosinophilic material deposition within a dilated Bowman’s space (star). Note a dilated renal tubule with cellular debris in the lumen [H&E]. (**E** and **F**) Kidney: Dystrophic calcification (mineralization) within the medullary region (arrows) [H&E]

**Fig. 3:**
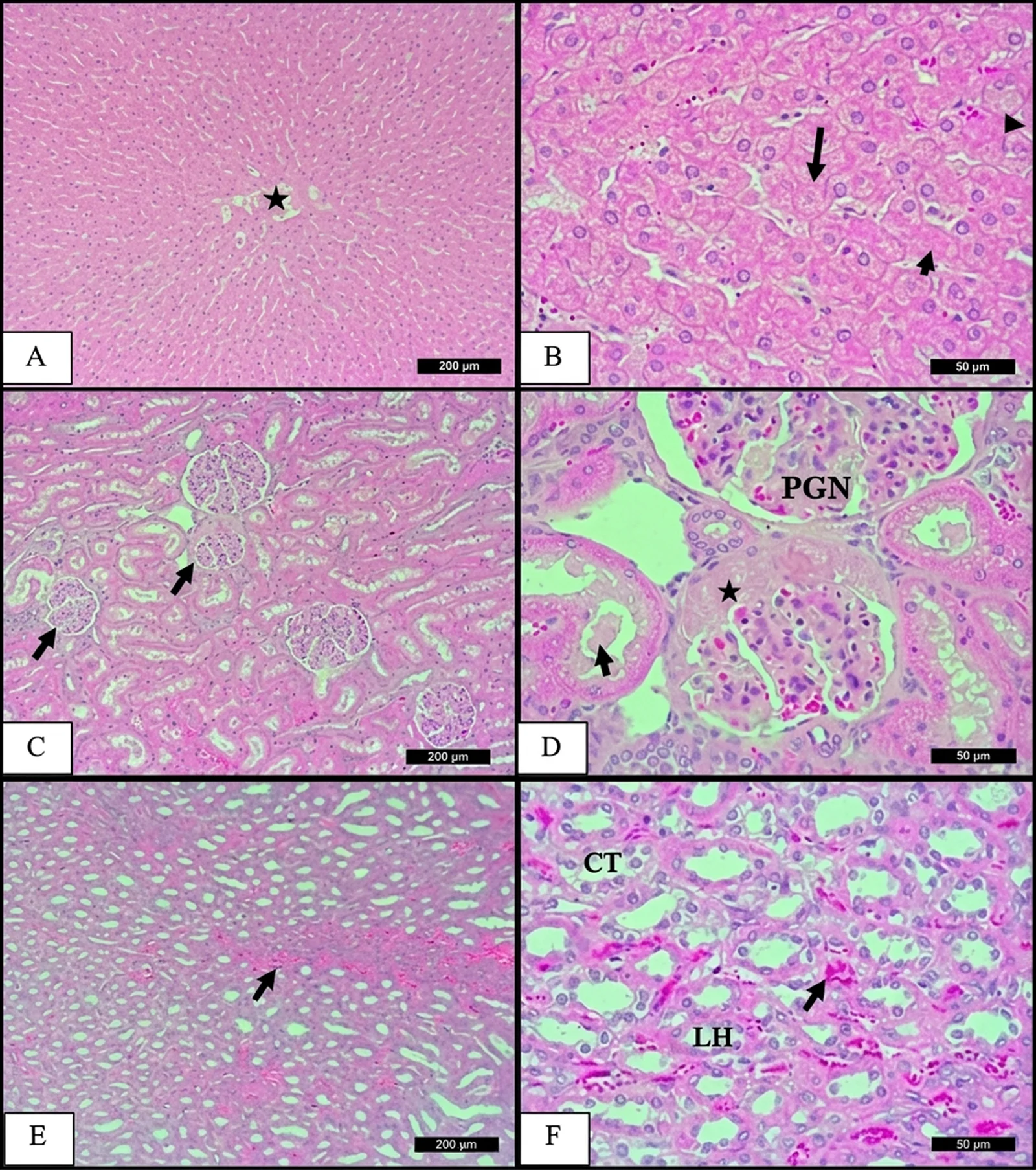
Photomicrographs of liver and kidneys parenchyma of Soinga lambs fed Sisal mucilage silage with ground corn additive. (**A**) Liver: Centrilobular region showing cords of hepatocytes with homogeneously pale eosinophilic cytoplasm surrounding the central vein (star) [H&E]. (**B**) Liver: High-magnification view showing necrotic hepatocytes (large arrow). Ballooning degeneration of hepatocytes is evident, characterized by peripherally displaced nuclei and intra-cytoplasmic accumulation of eosinophilic material suggestive of Mallory-Denk bodies (arrowhead). Note a necrotic hepatocyte exhibiting hyaline degeneration with a distinct Mallory-Denk body (small arrow) [H&E]. (**C**) Kidney: Atrophic glomeruli (arrow) alongside dilated renal tubules containing intraluminal cellular debris (arrow) [H&E]. **(D**) Kidney: High-magnification view of proliferative glomerulonephritis (PGN) adjacent to an atrophic glomerulus with crescent formation and deposition of eosinophilic material within a dilated Bowman’s space (star) [H&E]. (**E** and **F**) Kidney: Mild congestion (arrow) within the medullary region (CT: collecting tubule; LH: loop of Henle) [H&E]

Tha use of SMS, with or without additives, induced widespread histopathological alterations in both the liver (Table 3) and kidneys (Table 4) of lambs (Figs. 1, 2 and 3).

**Table 3:** Frequency and intensity of histopathological lesions in the liver of lambs fed sisal mucilage silage, with or without moisture-absorbing additives

Lesions	Treatments		
	SMS^a^	SMS-WB^b^	SMS-GC^c^
Absolute and relative frequency			
Sinusoidal atrophy	4/6 (66.6%)	2/6 (33.3%)	1/6 (16.6%)
Sinusoidal dilation	1/6 (16.6%)	1/6 (16.6%)	2/6 (33.3%)
Atrophy of hepatocellular cords	2/6 (33.3%)	3/6 (50.0%)	3/6 (50.0%)
Congestion of the hepatic vein	2/6 (33.3%)	2/6 (33.3%)	5/6 (83.3%)
Congestion of the central vein	6/6 (100%)	2/6 (33.3%)	5/5 (83.3%)
Mallory-Denk bodies	5/6 (83.3%)	4/6 (66.6%)	5/6 (83.3%)
Periportal hepatitis	2/6 (33.3%)	1/6 (16.6%)	1/6 (16.6%)
Hepatocellular hyperplasia	2/6 (33.3%)	1/6 (16.6%)	1/6 (16.6%)
Hepatocellular hypertrophy	5/6 (83.3%)	4/6 (66.6%)	2/6 (33.3%)
Macrovesicular steatosis	1/6 (16.6%)	0/6 (0%)	6/6 (100%)
Microvesicular steatosis	4/6 (66.6%)	5/6 (83.3%)	6/6 (100%)
Coagulative necrosis of hepatocytes	5/6 (83.3%)	6/6 (100%)	6/6 (100%)
Pericentrillobular necrosis of hepatocytes	3/6 (50.0%)	0/6 (0%)	3/6 (50.0%)
Intensity			
Coagulative necrosis of hepatocytes	+++	++	++
Microvesicular steatosis	+++	+	+++
Macrovesicular steatosis	+	-	++

^a^sisal mucilage silage without additives; ^b^sisal mucilage silage with wheat bran; ^c^sisal mucilage silage with ground corn. absent (-), mild (+), moderate (++), or severe (+++)

**Table 4:** Frequency and intensity of histopathological lesions in the kidneys of lambs fed sisal mucilage silage, with or without moisture-absorbing additives

Lesions	Treatments		
	SMS^a^	SMS-WB^b^	SMS-GC^c^
Absolute and relative frequency			
Glomerular atrophy	6/6 (100%)	3/6 (50.0%)	6/6 (100%)
Dystrophic calcification	4/6 (66.6%)	4/6 (66.6%)	4/6 (66.6%)
Cortical congestion	2/6 (33.3%)	1/6 (16.6%)	1/6 (16.6%)
Medullary congestion	4/6 (66.6%)	1/6 (16.6%)	3/6 (50.0%)
Corticomedullary congestion	4/6 (66.6%)	5/6 (83.3%)	3/6 (50.0%)
Glomerular congestion	2/6 (33.3%)	1/6 (16.6%)	1/6 (16.6%)
Tubular congestion	1/6 (16.6%)	0/6 (0%)	1/6 (16.6%)
Intraluminal tubular cellular debris	2/6 (33.3%)	5/6 (83.3%)	6/6 (100%)
Hydropic degeneration	5/6 (83.3%)	5/6 (83.3%)	6/6 (100%)
Interstitial edema	6/6 (100%)	1/6 (16.6%)	3/6 (50.0%)
Arterial wall thickening	4/6 (66.6%)	2/6 (33.3%)	4/6 (66.6%)
Membranoproliferative glomerulonephritis	2/6 (33.3%)	3/6 (50.0%)	1/6 (16.6%)
Proliferative glomerulonephritis	5/6 (83.3%)	6/6 (100%)	6/6 (100%)
Intensity			
Proliferative glomerulonephritis	++/+++	++/+++	+
Glomerular atrophy	++/+++	+	+

^a^sisal mucilage silage without additives; ^b^sisal mucilage silage with wheat bran; ^c^sisal mucilage silage with ground corn. mild (+), moderate (++), severe (+++), or moderate-to-severe (++/+++)

Hepatocellular coagulative necrosis was a pervasive finding across all groups (≥ 83.3%), peaking at 100% (6/6) in the additive-treated groups; however, its severity was down-graded from severe (+++) in the unadditived SMS group to moderate (++) in the SMS-WB and SMS-GC groups. Steatosis was predominantly microvesicular, affecting 100% (6/6) of SMS-GC lambs with severe (+++) intensity, whereas wheat bran (SMS-WB) markedly mitigated its severity to mild (+) and completely prevented macrovesicular steatosis (0%) and pericentral necrosis (0%) (Table 3).

On the other hand, the SMS-GC group exhibited a 100% frequency of moderate (++) macrovesicular steatosis. Centrilobular vein congestion was highest in SMS (100%) and SMS-GC (83.3%), dropping to 33.3% in SMS-WB. Other cellular features, such as Mallory-Denk bodies (MDB) (≥ 66.6%), hepatocellular hypertrophy (≥ 33.3%), and sinusoidal atrophy (≥ 16.6%), occurred across all treatments, while periportal hepatitis and hyperplasia remained low (≤ 33.3%) (Table 3).

Glomerular damage was marked by proliferative glomerulonephritis, which occurred at high frequencies (≥ 83.3%) across all groups. However, it was markedly more aggressive (moderate-to-severe, ++/+++) in the SMS and SMS-WB groups compared to the mild (+) changes seen in SMS-GC lambs. Glomerular atrophy was universally observed (100%) in the SMS and SMS-GC groups, but was reduced to 50% in SMS-WB. Its intensity was moderate-to-severe (++/+++) in pure SMS, but mitigated to mild (+) by both additives. Membranoproliferative glomerulonephritis was highest in the SMS-WB group (50%) (Table 4).

Tubular damage included hydropic degeneration (≥ 83.3%) and a treatment-dependent accumulation of intraluminal cellular debris, which peaked in the SMS-GC group (100%). Interstitial edema was prominent in pure SMS (100%) but effectively suppressed in SMS-WB (16.6%). Medullary dystrophic calcification remained constant at 66.6% across all groups. Furthermore, corticomedullary congestion peaked in the SMS-WB group (83.3%), while arterial wall thickening was lower in SMS-WB (33.3%) than in the other treatments (66.6%) (Table 4).

## Discussion

SMS-WB was the only diet that did not induce macrosteatosis and centrilobular necrosis in the sheep. Such alterations, observed in the hepatic tissue of animals in the other experimental groups are suggestive of toxicosis. Centrilobular hepatocyte necrosis is common in hepatotoxic conditions and occurs due to the high enzymatic activity of hepatocyte oxidases. These enzymes convert inactive exogenous compounds into toxic metabolites, suggesting hepatic damage associated with the diets provided (Cullen 2007).

The inclusion of WB as an additive reduced microsteatosis from severe to mild, thus demonstrating a hepatoprotective effect. This result agrees with the findings of Junejo et al. (2019), who attributed this response to the fibrous structure of WB, which reduces sterol and cholesterol absorption, limits hepatic triglyceride accumulation, and promotes the production of beneficial short-chain fatty acids and bioactive polyphenols through microbial fermentation, thereby attenuating steatosis and hepatocellular damage.

On the other hand, the SMS-GC diet exacerbated hepatic lesions, resulting in a 100% incidence and high severity of microvesicular steatosis. These findings may be related to the higher NFC content of the diet containing this silage (Table 2), which may have overloaded hepatic lipid metabolism. In addition, plants containing saponins are toxic to ruminants, causing gastroenteritis, diarrhea, and even hepatic and renal degeneration (Wina et al. 2005). Silva et al. (2021) and Dias et al. (2022), while investigating the hepatic histopathology of goats and lambs fed cactus cladodes, respectively, reported coagulative hepatocyte necrosis, attributing this finding to the presence of antinutritional or toxic factors in the diets.

At the hepatic level, coagulative necrosis was the most frequently observed lesion. The primary cause of this type of necrosis is ischemia, resulting, for example, from the obstruction of blood vessels supplying the tissue (Kumar et al. 2010; Canepa et al. 2024). In the present study, centrilobular vein congestion and sinusoidal capillary atrophy, both of which may impair hepatic blood flow, were more frequently observed in lambs fed unadditive-treated SMS than in those receiving additive-treated silages.

Although MDBs were observed across all experimental groups, their presence was more pronounced in the SMS and SMS-GC groups than in the SMS-WB group. These abnormal protein aggregates are associated with various hepatopathies, including metabolic dysfunction-associated steatotic liver disease in humans (Zatloukal et al. 2007; Denk et al. 2024). Therefore, quantifying the alcohol content of the silages could elucidate the pathogenesis of these cytoplasmic hyaline inclusions. Nevertheless, despite the classic association between MDBs and severe hepatic disease, the lambs in the current study manifested no clinical signs of metabolic disorders.

The atrophy of sinusoidal capillaries observed in lambs fed SMS (without additive) was likely driven by mechanical compression resulting from concurrent hepatocyte hyperplasia and hypertrophy. Consistently, the hepatic parenchyma of animals in this group exhibited a higher frequency of both increased cell numbers and enlarged hepatocyte size compared to the additive-treated groups (Table 3).

Incorporating corn as an additive likely mitigated the daily ingested dose of hepatotoxic secondary metabolites via a dilution effect. This reduction attenuated direct cellular damage to hepatocytes, which was clinically evidenced by a decrease in the severity of the main histopathological lesions (Table 3).

The most frequent histopathological lesions in the kidneys of lambs fed SMS with or without additives were proliferative glomerulonephritis, glomerular atrophy, and hydropic degeneration (Table 4). Proliferative glomerulonephritis is typically associated with inflammatory glomerular injury mediated by immune complex deposition. In sheep, this condition can occur spontaneously as a nonspecific inflammatory alteration frequently observed during necropsy, often lacking clinical significance (Jones et al. 2012).

The severity of renal glomerular atrophy was lower in lambs fed SMS supplemented with corn or wheat, although the incidence of this lesion reached 100% in the SMS-GC group. As occurs in most forms of chronic glomerular disease, tubular atrophy typically follows the progressive loss of renal function (Wasserstein 1997). In the current study, however, the incorporation of these moisture-absorbing additives effectively protected the renal parenchyma, thereby mitigating functional decline.

A decreased frequency of kidney lesions, including glomerular atrophy, interstitial edema, arterial wall thickening, and medullary congestion, was noted in lambs fed the SMS-WB diet, although an increased frequency of corticomedullary congestion was concurrently detected (Table 4). Interstitial congestion has been previously reported in the kidneys of lambs following the administration of *Yucca schidigera* (Roetzl) juice, with saponins identified as the specific agents responsible for the resulting nephrotoxicity (Wisløff et al. 2008).

Calcification was observed in the renal parenchyma of lambs across all experimental groups, indicating that renal mineralization stands as an intrinsic and direct pathological effect of *Agave sisalana* mucilage that is insensitive to the tested additives. Plants of the genus *Agave* are notably rich in calcium and calcium oxalate crystals (Salinas et al. 2001; Santos et al. 2009; Bernardino-Nicanor et al. 2012; León-Martínez et al., 2022). Upon glomerular filtration, these oxalates trigger chronic mechanical and biochemical irritation within the renal tubular epithelium, a mechanism strongly corroborated in this study by the high frequency of intraluminal cellular debris. Consequently, these damaged epithelial cells suffer an uncontrolled influx of calcium into the cytosol, accumulating in the mitochondria and driving dystrophic medullary calcification (Newman 2012).

Oxalate-induced renal lesions, characterized by proliferative glomerulonephritis, hydropic degeneration, dystrophic calcification, glomerular atrophy, corticomedullary congestion, and even tubular cell necrosis, have been well-documented in sheep consuming cactus cladodes-based diets (Barboza et al. 2019; Usman et al. 2022).

Despite the hepatic and renal alterations induced by the additive-treated silages, lambs in these groups exhibited higher final body weights compared to those fed the without additive silage (33.00 kg for SMS-WB and 32.82 kg for SMS-GC vs. 30.26 kg for SMS; *P* = 0.0202), as reported by Sousa (2019) in a companion study. Overall, despite significant subclinical tissue damage, animal growth remained high. This outcome was likely influenced by the short trial duration, highlighting the need for future long-term studies.

These findings underscore the critical relevance of incorporating moisture-absorbing additives to enhance the nutritional value and productive viability of sisal mucilage silage, effectively offsetting subclinical tissue challenges by promoting superior animal performance. Therefore, while sisal mucilage silage exhibits intrinsic hepatorenal toxicity in lambs, the use of moisture-absorbing additives represents a vital mitigation strategy. Wheat bran serves as an effective hepatoprotector, whereas ground corn provides superior glomerular preservation. Such nutritional interventions are crucial to enable the safe use of sisal by-products in ruminant nutrition without compromising animal health and performance. Ultimately, we acknowledge some limitations of the present study: (a) bioactive compounds such as saponins and oxalates were not quantified in the fresh mucilage, additives or the final silages; and (b) the study lacks a combined additive group (SMS + WB + GC). Thus, we encourage future research to address these gaps.

## Data Availability

The data that support the findings of this study are available from the corresponding author upon reasonable request.
